# Improved Carotenoid Processing with Sustainable Solvents Utilizing *Z*-Isomerization-Induced Alteration in Physicochemical Properties: A Review and Future Directions

**DOI:** 10.3390/molecules24112149

**Published:** 2019-06-07

**Authors:** Masaki Honda, Hakuto Kageyama, Takashi Hibino, Yelin Zhang, Wahyu Diono, Hideki Kanda, Ryusei Yamaguchi, Ryota Takemura, Tetsuya Fukaya, Motonobu Goto

**Affiliations:** 1 Faculty of Science & Technology, Meijo University, Shiogamaguchi, Tempaku-ku, Nagoya 468-8502, Japan; kageyama@meijo-u.ac.jp (H.K.); hibino@meijo-u.ac.jp (T.H.); 2 Department of Materials Process Engineering, Nagoya University, Furo-cho, Chikusa-ku, Nagoya 464-8603, Japan; zhang.yelin@c.mbox.nagoya-u.ac.jp (Y.Z.); wahyudiono@b.mbox.nagoya-u.ac.jp (W.D.); kanda.hideki@material.nagoya-u.ac.jp (H.K.); 3 Technical Center, Nagoya University, Furo-cho, Chikusa-ku, Nagoya 464-8603, Japan; yamaguchi.ryusei@i.mbox.nagoya-u.ac.jp; 4 Innovation Division, Kagome Company, Limited, Nishitomiyama, Nasushiobara 329-2762, Japan; Ryota_Takemura@kagome.co.jp; 5 Institutes of Innovation for Future Society, Nagoya University, Furo-cho, Chikusa-ku, Nagoya 464-8603, Japan

**Keywords:** lycopene, β-carotene, astaxanthin, *E*/*Z*-isomerization, solubility, crystallinity, extraction, emulsification, micronization, supercritical CO_2_

## Abstract

Carotenoids—natural fat-soluble pigments—have attracted considerable attention because of their potential to prevent of various diseases, such as cancer and arteriosclerosis, and their strong antioxidant capacity. They have many geometric isomers due to the presence of numerous conjugated double bonds in the molecule. However, in plants, most carotenoids are present in the all-*E*-configuration. (all-*E*)-Carotenoids are characterized by high crystallinity as well as low solubility in safe and sustainable solvents, such as ethanol and supercritical CO_2_ (SC-CO_2_). Thus, these properties result in the decreased efficiency of carotenoid processing, such as extraction and emulsification, using such sustainable solvents. On the other hand, *Z*-isomerization of carotenoids induces alteration in physicochemical properties, i.e., the solubility of carotenoids dramatically improves and they change from a “crystalline state” to an “oily (amorphous) state”. For example, the solubility in ethanol of lycopene *Z*-isomers is more than 4000 times higher than the all-*E*-isomer. Recently, improvement of carotenoid processing efficiency utilizing these changes has attracted attention. Namely, it is possible to markedly improve carotenoid processing using safe and sustainable solvents, which had previously been difficult to put into practical use due to the low efficiency. The objective of this paper is to review the effect of *Z*-isomerization on the physicochemical properties of carotenoids and its application to carotenoid processing, such as extraction, micronization, and emulsification, using sustainable solvents. Moreover, aspects of *Z*-isomerization methods for carotenoids and functional difference, such as bioavailability and antioxidant capacity, between isomers are also included in this review.

## 1. Introduction

**Figure 1:**
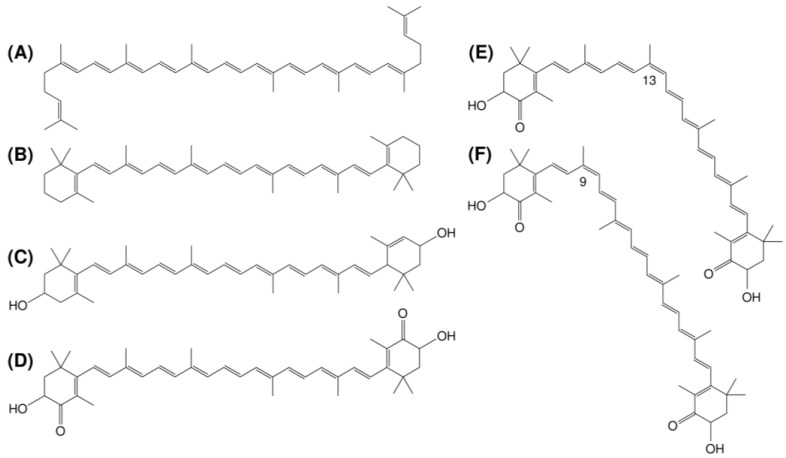
Chemical structures of (**A**) (all-*E*)-lycopene, (**B**) (all-*E*)-β-carotene, (**C**) (all-*E*)-lutein, (**D**) (all-*E*)-astaxanthin, (**E**) (13*Z*)-astaxanthin, and (**F**) (9*Z*)-astaxanthin.

Carotenoids are a class of lipid-soluble pigments responsible for the colors of plants, animals, and microorganisms [1,2,3,4]. Since the first structural elucidation of β-carotene by Kuhn and Karrer in the 1930s, approximately 1100 natural carotenoids have been reported so far [5]. Carotenoids can be classified into the following two groups based on their chemical composition: (1) carotenes, nonoxygenated molecules such as lycopene and β-carotene and (2) xanthophylls, molecules containing oxygen such as lutein and astaxanthin. (Figure 1) [4,6]. The daily consumption of carotenoid-rich foods, such as fruits and vegetables, is considered to be beneficial for human health because of their high antioxidant, anticancer, and antiatherosclerotic activities [7,8,9]. As carotenoids contain multiple conjugated double bonds, numerous geometric isomers are theoretically possible. While carotenoids in plants are accumulated predominantly as the all-*E*-configuration (Figure 1A–D), *Z*-isomers of carotenoids (Figure 1E,F) exist in abundance in the human body and in processed foods. For example, more than 50 and 30% of total lycopene are present as *Z*-isomers in human blood plasma and processed tomato products such as tomato sauce and tomato soup, respectively [10,11,12].

Commercially available carotenoids are obtained by chemical syntheses or extraction from plants, photosynthetic bacteria, and microalgae. Generally, these carotenoids are in the all-*E*-configuration and the isomers are characterized by high crystallinity and low solubility in solvents [13,14]. Most carotenoid processing, such as extraction, micronization, and emulsification, employs a mediator—an organic solvent—to increase the processing efficiency. However, because of the physicochemical properties of (all-*E*)-carotenoids, processing efficiencies are low. Moreover, in recent years, there has been increased demand for the use of safe and sustainable solvents such as ethanol and supercritical CO_2_ (SC-CO_2_) for the processing of food components including carotenoids, i.e., environmentally benign processing using sustainable solvents is a topic of growing interest in both the research community and the food industry because of the growing awareness of the impact of solvents on energy usage, pollution, and their contribution to climate change and air quality [15,16,17]. However, since (all-*E*)-carotenoids have very low solubility in ethanol and SC-CO_2_ [18,19,20,21], toxic organic solvents are used in many cases. Very recently, several studies demonstrated that *Z*-isomerization of carotenoids induces alteration in physicochemical properties, such as crystallinity and solubility. Namely, solubility in solvents including SC-CO_2_ was dramatically improved and crystallinity was reduced by *Z*-isomerization. In addition, application of these alterations in carotenoid processing using the above safe and sustainable solvents has attracted attention. For example, *Z*-isomerization pretreatment significantly improved the extraction efficiency of lycopene contained in tomatoes and gac (*Momordica cochinchinensis* Spreng.) aril using organic solvents and SC-CO_2_ [18,19].

In this review, the effect of *Z*-isomerization on the physicochemical properties of carotenoids and recent researches on carotenoid processing technology exploiting these characteristics are summarized and discussed. In addition, we also outline the typical methods for *Z*-isomerization of carotenoids and alterations in the bioavailability and functionality of carotenoids by *Z*-isomerization. Ample studies have demonstrated that *Z*-isomerization of carotenoids results in changes to bioavailability and antioxidant capacity, e.g., *Z*-isomers of lycopene and astaxanthin have greater bioavailability and show a higher antioxidant capacity than the all-*E*-isomers [22,23,24,25]. Thus, it is important to have a thorough understanding of the impact of *E*/*Z*-isomerization on functional changes of carotenoids.

## 2. Typical Methods for *Z*-Isomerization of Carotenoids

As the method for *Z*-isomerization of carotenoids, heat treatment, microwave treatment, light irradiation, electrolysis treatment, and catalytic treatment have been well documented to date (Table 1). (all-*E*)-Carotenoids, e.g., lycopene and astaxanthin, were efficiently isomerized to the *Z*-isomers by heating in organic solvents, especially alkyl halides such as dichloromethane (CH_2_Cl_2_) and dibromomethane (CH_2_Br_2_) [26,27,28]. The all-*E*-isomers were also thermally *Z*-isomerized in the presence of vegetable oils, animal fats, and SC-CO_2_ [12,21]. These results indicate that for *Z*-isomerization of (all-*E*)-carotenoids, it is important that the carotenoid is in a dissolved state. Microwave heating also promoted *Z*-isomerization [29,30,31,32], and several studies indicated the increased efficiency compared to conventional heating [29,30]. In the microwave treatment of (all-*E*)-lycopene-rich tomato oleoresin, the total *Z*-isomer content reached 65.9 ± 2.7% with 4-min irradiation at 2450 MHz frequency and 500 W power, and the temperature of the oleoresin reached 136.7 ± 6.6 °C at that time, while the total *Z*-isomer content with conventional oil bath heating at 140 °C for 5 min was 50.8 ± 3.2% [29]. Light irradiation also caused *E*/*Z*-isomerization of carotenoids. When carotenoids were directly exposed to light, *Z*-isomers of carotenoids isomerized to the all-*E*-isomers [33,34]. On the other hand, when light irradiation was carried out with photosensitizers, such as chlorophyll *a* and erythrosine, *Z*-isomerization of (all-*E*)-carotenoids was promoted [35,36]. For example, when purified (all-*E*)-lycopene dissolved in hexane in the presence of erythrosine was irradiated at 480–600 nm for 1 h, the proportion of lycopene *Z*-isomers reached over 80% [36]. A few studies have demonstrated that electrolysis treatment promoted *Z*-isomerization of (all-*E*)-carotenoids such as β-carotene and canthaxanthin [37,38]. This electrochemical method shows very high efficiency and can prevent thermal degradation of carotenoids, e.g., approximately 50% of (all-*E*)-canthaxanthin was converted to the *Z*-isomers during 4–6 min of bulk electrolysis at 4 °C [37]. Catalytic *Z*-isomerization of (all-*E*)-carotenoids have been traditionally conducted using iodine [39,40,41]. More recently, it was reported that disulfide compounds [29,42,43], isothiocyanates, carbon disulfide [42], iron(III) chloride [44], titanium tetrachloride [45], and iodine-doped titanium dioxide [46] induced *Z*-isomerization of carotenoids. For example, when iron(III) chloride, which is usually used as a food additive for iron fortification, was used, greater isomerization (79.9%) could be attained by a 3-h reaction at 60 °C, with almost no degradation of lycopene [44]. Although catalyst utilization for carotenoid isomerization is a very efficient method, most catalysts, such as iodine and heavy metals, exert a negative impact on the human body and the environment. Hence, in industrial view, the discovery and use of low toxicity and environmentally sustainable catalysts, e.g., plant-derived natural catalysts such as disulfide compounds and isothiocyanates, will be of great importance [29,42,43,47,48,49]. As shown in Table 1, each *Z*-isomerization method has several advantages and disadvantages; consequently, it is necessary to select the appropriate *Z*-isomerization method according to the circumstances.

## 3. Effect of *Z*-Isomerization on Bioavailability and Functionality of Carotenoids

Ample studies have addressed the alterations in bioavailability and functionality, such as antioxidant, anticancer, and antiatherosclerotic activities, of carotenoids by *Z*-isomerization (Table 2). Further, the alterations differed among carotenoids. *Z*-Isomers of lycopene and astaxanthin showed greater bioavailability than the all-*E*-isomers [22,23,25,50,51,52,53]. For example, investigation of the effect of red tomato juice (90% all-*E*-isomer lycopene) and *tangerine* tomato juice (94% *Z*-isomer lycopene) ingestion on plasma lycopene concentrations revealed that lycopene from *tangerine* tomato juice showed approximately 8.5-fold greater bioavailability than lycopene from red tomato juice [22]. On the other hand, some carotenoid *Z*-isomers, such as β-carotene and lutein, may be less bioavailable than the all-*E*-isomers [54,55,56,57,58,59,60,61,62]. In general, the bioavailability of carotenoids is very low because they are strongly bound to the food matrix and because of their physicochemical characteristics, such as low solubility, high crystallinity, and lipophilicity [4,13,14]. Thus, to improve the bioavailability of carotenoids contained in fruits and vegetables, physical treatments, such as high-pressure homogenization and ultrasound treatment, have been traditionally studied [63]. In some carotenoids, such as lycopene and astaxanthin, by combining chemical approaches such as *Z*-isomerization treatment and the above physical approaches, it may be possible to further improve the bioavailability.

Depending on the assay method, many studies have reported that *Z*-isomers of carotenoids have equal or higher antioxidant capacity compared with the all-*E*-isomers [24,25,46,63,64,65,66,67,68,69,70]. Böhm et al. (2002) [64] reported that *Z*-isomers of lycopene exhibited approximately 1.2 times higher antioxidant capacities than the all-*E*-isomer in the Trolox equivalent antioxidant capacity (TEAC) assay. In heme-induced peroxidation of linoleic acid in mildly acidic emulsions, which mimics postprandial lipid oxidation in the gastric compartment (MbFe^III^-LP) assay, (5*Z*)-lycopene showed approximately 3 times higher antioxidant capacity than the all-*E*-isomer [24]. In contrast, when antioxidant capacity was evaluated by the TEAC assay, (9*Z*)-zeaxanthin exhibited lower capacity than the all-*E*-isomer [64]. The degree of antioxidant capacity varied among *Z*-isomers, e.g., that of lutein isomers was higher in the order of 13*Z*-isomer > 9*Z*-isomer > all-*E*-isomer with the ferric reducing antioxidant power (FRAP) assay [62]. Carotenoid *Z*-isomers are likely to be superior to the all-*E*-isomers in preventative effects on atherosclerosis, cancer, and inflammatory [71,72,73,74,75,76]. For example, (9*Z*)-β-carotene contained in the microalgae *Dunaliella* sp. showed higher antiatherogenic [71] and antiatherosclerotic [72,73] activities than the all-*E*-isomer in mouse experiments. (9*Z*)-Canthaxanthin isolated from *Dietzia* sp. exhibited higher proapoptotic activity than the all-*E*-isomer in THP-1 macrophages [74]. Nakazawa et al. (2009) [75] reported that *Z*-isomers of fucoxanthin had greater anticancer activity than the all-*E*-isomer in human promyelocytic leukemia (HL-60) and colon cancer (Caco-2) cells. Very recently, Yang et al. (2019) [76] showed that *Z*-isomers of astaxanthin, especially the 9*Z*-isomer, exhibited greater antiinflammatory effect than the all-*E*-isomer by downregulating proinflammatory cytokines COX-2 and TNF-α gene expression, which was evaluated in a Caco-2 cell monolayer model. As another notable functional change by carotenoid *Z*-isomerization, *Z*-isomers of β-carotene, which is a very important retinol precursor with a high conversion rate, showed lower conversion efficiencies to retinol than the all-*E*-isomer [77,78]. The antiaging activity would also differ among astaxanthin isomers. Namely, the median lifespan of *Caenorhabditis elegans* fed with 9-*Z*-, 13-*Z*-, and all-*E*-isomers was observed to increase by 59.39%, 24.99%, and 30.43%, respectively [79]. Moreover, Fenni et al. (2019) [80] reported that lycopene isomers exert similar biological functions in adipocytes, linked to their ability to transactivate PPARγ. Since *Z*-isomerization had “positive” or “negative” effects on the bioavailability and functionality of carotenoids (Table 2), it is important to have a detailed understanding of the impact of *E*/*Z*-isomerization on corresponding functional changes. 

## 4. Effect of *Z*-Isomerization on Physicochemical Properties of Carotenoids

The *Z*-isomerization of (all-*E*)-carotenoids induces change in physicochemical properties such as color, solubility, crystallinity, melting point, and stability. *Z*-Isomerization of carotenoids resulted in a shift in absorption to a shorter wavelength and a reduction in the molar extinction coefficient and color value [27,46,82,83]. For example, Jing et al. (2012) [83] reported that maximum absorption wavelengths of (all-*E*)-, (9*Z*)-, and (13*Z*)-β-carotene were 451.4, 446.4, and 439.1 nm, respectively. The molar extinction coefficients of (all-*E*)-, (9*Z*)-, and (13*Z*)-lycopene at the maximum absorption wavelengths were 182 × 10^3^, 164 × 10^3^, and 137 × 10^3^ M^−1^ cm^−1^, respectively [27]. In fact, tomatoes rich in (all-*E*)-lycopene show a red color, whereas tomatoes rich in the *Z*-isomers, known as *tangerine* tomatoes, show an orange color [22].

Several studies reported that *Z*-isomers of carotenoids had much higher solubility than the all-*E*-isomers in organic solvents, oils, and SC-CO_2_ [13,14,18,19,20,21,84,85]. Although the solubility of (all-*E*)-lycopene in ethanol, acetone, ethyl acetate, and hexane was 0.6, 42.7, 145.3, and 25.6 mg/mL, respectively, that of lycopene containing 75.6% *Z*-isomers was 2401.7, 3702.9, 3961.1, and 3765.2 mg/mL, respectively [13]. Namely, in the case of ethanol, which is frequently used for food processing such as extraction and purification, the solubility of lycopene *Z*-isomers was over 4000 times higher than that of the all-*E*-isomer. Also, in SC-CO_2_, the solubility of (9*Z*)-β-carotene was nearly four times higher than that of the all-*E*-isomer [84], and lycopene *Z*-isomers also showed higher solubility than the all-*E*-isomer [19,21]. The increased solubility of carotenoids by *Z*-isomerization is likely to be associated with changes in bioavailability. Generally, carotenoids are absorbed from the duodenum and prior to the absorption they are incorporated into bile acid micelles [86]. Thus, since carotenoid *Z*-isomers may have higher solubility in bile acid than all-*E*-isomers, they are preferentially incorporated into enterocytes and show higher bioavailability [51,87]. On the other hand, *Z*-isomers of β-carotene exhibit lower bioavailability in humans than the all-*E*-isomer [54,55,56,57,58]. Several proteins, which are temporarily present at the apical membrane of the duodenum, mediate selective carotenoid uptake [86]. Therefore, β-carotene *Z*-isomers may be efficiently incorporated into bile acid micelles due to their high solubility, but may have lower transport efficiency in the duodenum than the all-*E*-isomer. In vitro experiments using Caco-2 cells strongly support the above hypothesis. Namely, *Z*-isomers of lycopene and astaxanthin showed higher cellular uptake efficiency than the all-*E*-isomers [25,52], while the opposite result was obtained for β-carotene [61]. Similarly, Yang et al. (2018) [62] reported that in vitro experiments using a digestion model shown higher bioaccessibility of lutein *Z*-isomers than the all-*E*-isomer, while a Caco-2 cell monolayer model revealed lower bioavailability.

*Z*-Isomerization of carotenoids affects the crystallinity. Murakami et al. (2017) [13] and Honda et al. (2018) [14] experimentally revealed that increases in the *Z*-isomer content of lycopene, β-carotene, and astaxanthin was related to a reduction in crystallinity, i.e., scanning electron microscopy (SEM), differential scanning calorimetry (DSC), and powder X-ray diffraction (XRD) analyses clearly demonstrated that (all-*E*)-carotenoids were present in a crystal state, while *Z*-isomers were present in an amorphous state. Carotenoids have multiple conjugated double bonds in the molecule, resulting in strong π–π stacking interactions between molecules. For this reason, carotenoids have high crystallinity. However, the presence of *Z*-isomers is suggested to lead to enormous steric hindrance and decrease the potential attractive π–π forces, thus affecting the crystallinity [13,88]. Generally, carotenoids in fresh plants occur predominantly in the (all-*E*)-configuration, and (all-*E*)-carotenoids are present in the crystal state. On the other hand, some plants, such as *tangerine* tomato and peach palm (*Bactris gasipaes* Kunth), contain high amounts of carotenoid *Z*-isomers that are present in an oily aggregate form [22,89]. Similarly, 9*Z*-isomer-rich β-carotene contained in *Dunaliella* was in the oily form [90].

The melting point of carotenoids was altered by *Z*-isomerization, i.e., increases in the *Z*-isomer content were associated with a lower melting point [13,14,85,91]. For example, the melting point of (all-*E*)-lycopene and lycopene containing 23.8, 46.9, and 75.6% *Z*-isomers was 174.4, 173.7, 170.0, and 162.3 °C, respectively, as measured by DSC [13].

The stability of carotenoids varies among isomers, i.e., (all-*E*)-carotenoids had higher stability than the *Z*-isomers. Several studies investigated the stability of carotenoid isomers using a Gaussian program and revealed that Gibbs free energy differed among the isomers [82,92,93,94]. For example, Takehara et al. (2015) [93] reported that the relative stability of lycopene isomers was in the following order; all-*E*-isomer ≈ 5*Z*-isomer > 9*Z*-isomer > 13*Z*-isomer > 15*Z*-isomer, and Guo et al. (2008) [94] reported that the relative stability of β-carotene isomers was in the following order; all-*E*-isomer > 9*Z*-isomer > 13*Z*-isomer > 15*Z*-isomer > 7*Z*-isomer ≈ 11*Z*-isomer. Murakami et al. (2018) [33] experimentally confirmed the above for lycopene. Furthermore, they investigated the stability of lycopene isomers against light irradiation, and the stability was in the following order; all-*E*-isomer ≈ 5*Z*-isomer > 9*Z*-isomer > 13*Z*-isomer > multi-*Z*-isomers. As for lycopene *Z*-isomers, the 5*Z*-isomer showed the highest stability against heat and light. In addition, (5*Z*)-lycopene would have higher antioxidant capacity [24] and bioavailability [95] compared with the all-*E*-isomer and possibly the 9*Z*- and 13*Z*-isomers. Therefore, it is important to develop a facile procedure for lycopene isomerization from the all-*E*-isomer to the 5*Z*-isomer.

The differences in physicochemical properties between (all-*E*)-carotenoids and *Z*-isomers are summarized in Table 3. A systematic understanding of these carotenoid properties is likely to be important in the analysis, processing, and so on.

## 5. Improvement of Carotenoid Processing Efficiency by *Z*-Isomerization

In recent years, due to the discovery of altered physicochemical properties of carotenoids by *Z*-isomerization, efforts to improve the efficiency of carotenoid processing by exploiting these alterations has attracted attention. In particular, carotenoid processing using a safe and sustainable solvent—SC-SO_2_—as a mediator is being actively studied. Since natural carotenoids, the all-*E*-isomer, exhibit very low solubility in SC-SO_2_, there is a high hurdle for its industrial use in carotenoid processing. However, utilizing alterations in the physical properties by *Z*-isomerization represents a breakthrough. In this section, we introduce recent studies of carotenoid processing (extraction, micronization, and emulsification) utilizing alterations in solubility and crystallinity of carotenoids by *Z*-isomerization.

### 5.1. Improvement of Carotenoid Extraction

**Figure 2:**
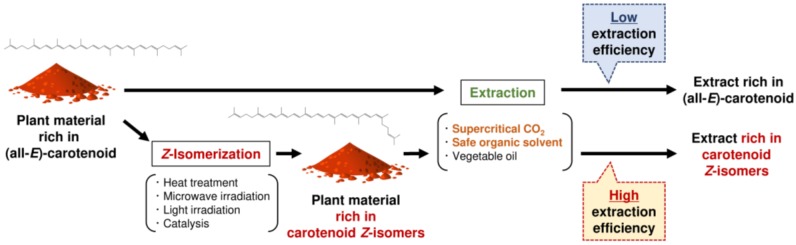
Schematic chart showing extraction of lycopene from plant material by solvents in the case of using (all-*E*)-lycopene and lycopene *Z*-isomers as the raw materials [18,19].

Generally, commercially available natural carotenoids, which are obtained from plants and microorganisms by solvent extraction and utilized for supplements, food colorants, and cosmetics, are very expensive [96,97,98,99]. This is because carotenoids in plants and microorganisms accumulate predominantly in the all-*E*-configuration, whose isomers have low solubility in solvents, resulting in very low extraction efficiencies. For example, extraction of lycopene from tomato pulp with ethanol and SC-CO_2_ showed a recovery of only 6.3 and 6.5%, respectively [19]. However, when the extractions were conducted after *Z*-isomerization treatment, the recovery was notably improved to 75.9 and 27.6%, respectively [19]. More specifically, the total *Z*-isomer content of lycopene in tomato pulp was 8.8%, whereas it increased to 75.7% by heating at 150 °C for 1 h with a small amount (1 wt%) of olive oil. After ethanol extraction of lycopene from the *Z*-isomer-rich tomato pulp, the obtained extract had a very high *Z*-isomer content (93.5%), while almost all lycopene in the extraction residue was the all-*E*-isomer. These results strongly indicated that lycopene *Z*-isomers have higher solubility in solvents than the all-*E*-isomer; thus, the extraction efficiency was improved. In addition, since the *Z*-isomer content of carotenoids in the obtained extract was improved by *Z*-isomerization pretreatment, the treatment is effective not only for the production of carotenoid concentrates but also for increasing the bioavailability and functionality of carotenoids (Figure 2). The improved extraction efficiency was also confirmed in gac (*M. cochinchinensis* Spreng.) aril [18]. Gac is a tropical vine originating from South and South-East Asia and belongs to the Cucurbitaceae family, and the aril (seed membrane) contains a very high amount of lycopene [100,101]. Since gac aril contains a large amount of oil (18–34% of dry weight) rich in lycopene, lycopene is often obtained by press extraction with the oil [102]. Although more than 90% of lycopene exists as the all-*E*-isomer in gac aril, the total *Z*-isomer content increased by 58.5% with microwave irradiation at 1050 W for 60 s. When lycopene was obtained by press extraction with gac oil from non-microwave pretreated and treated gac aril, lycopene contents in the obtained oils were 160.6 and 1365.9 mg/100 g, respectively. Thus, *Z*-isomers of carotenoids show higher solubility in oils than the all-*E*-isomer. Moreover, *Z*-isomerization pretreatment of gac aril was also effective for lycopene extraction using ethanol and SC-CO_2_. For example, when lycopene was extracted using SC-CO_2_ from the non-treated gac aril, the lycopene content in the extract was only 76.6 mg/100 g, whereas *Z*-isomerization pretreatment by microwave irradiation resulted in a lycopene content of 342.0 mg/100 g. As the extraction efficiency of carotenoids is improved by *Z*-isomerization pretreatment, the development of efficient *Z*-isomerization methods for carotenoids in plants is very important in the future. On the other hand, several plants and microalgae such as *tangerine* tomato and *Dunaliella* contain a high amount of carotenoid *Z*-isomers [22,71,72]. Thus, carotenoids should be efficiently extracted using these raw materials. In fact, Gamlieli-Bonshtein et al. (2002) [84] reported that (9*Z*)-β-carotene in *Dunaliella* exhibited nearly 4 times higher extraction efficiency by SC-CO_2_ than the all-*E*-isomer. Pretreatments of samples by physical and chemical approaches such as grinding, osmotic shock, bead-beating, high-pressure homogenization, and enzymatic treatment are effective in releasing carotenoids from complex matrices, and have been performed in basic and applied studies [103,104]. On the other hand, *Z*-isomerization pretreatment is a new technology reported very recently. By combing traditional physical and chemical pretreatments and *Z*-isomerization pretreatment, further improvement of carotenoid extraction can be expected. In addition, when the *Z*-isomerization pretreatment is used in combination with several extraction technique, such as pulsed electric field-assisted extraction, microwave-assisted extraction, and ultrasonic-assisted extraction, synergistic effects are expected [105,106,107,108,109].

### 5.2. Improvement of Carotenoid Micronization

**Figure 3:**
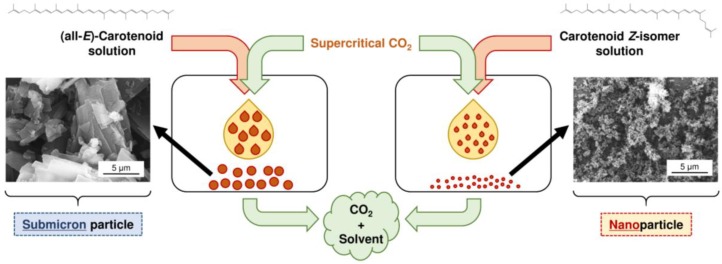
Schematic chart showing preparation of lycopene particles with supercritical CO_2_ (solution-enhanced dispersion by supercritical fluids), using (all-*E*)-lycopene and lycopene *Z*-isomers as the raw materials [117].

Ample studies have reported that micronization of carotenoids results in their increased bioavailability [110,111]. Generally, carotenoid micronization is conducted by milling, grinding, and chemical precipitation [112,113,114]. However, there are some concerns regarding the above conventional methods, as carotenoids are easily decomposed by friction heat and oxygen contact. In addition, when using chemical processes, toxic organic solvents may remain. Thus, in recent years, micronization of carotenoids using SC-CO_2_ has attracted increasing attention. Since CO_2_ is nontoxic and has a low critical temperature (*T*_c_ = 31.1 °C), it is suitable for heat-sensitive materials such as carotenoids, and SC-CO_2_ is easily separated from the products along with the toxic organic solvent [115,116]. To the best of our knowledge, improved micronization efficiency of carotenoids utilizing alterations in the physicochemical properties by *Z*-isomerization has been reported only for the method using SC-CO_2_ [117]. Particle micronization techniques using SC-CO_2_, supercritical antisolvent (SAS), solution-enhanced dispersion by supercritical fluids (SEDS), rapid expansion of supercritical solutions (RESS), gas antisolvent (GAS), supercritical fluid extraction of emulsions (SFEE), and particles from gas saturated solutions (PGSS) have been well-documented [118,119,120,121,122]. Several studies have examined the micronization of carotenoids using the above techniques; however, there was difficulty in obtaining nano-sized carotenoid particles [123,124,125]. For example, Tavares-Cardoso et al. (2009) [125] conducted micronization of (all-*E*)-β-carotene using a SAS process under various conditions; however, nano-sized β-carotene particles could not be obtained. This is likely because of the high crystallinity of carotenoids. On the other hand, Kodama et al. (2018) [117] successfully prepared nano-sized lycopene by SEDS precipitation using lycopene *Z*-isomers as the raw material. Namely, when using (all-*E*)-lycopene as the raw material, particles having an average size of 3.6 μm were obtained, whereas when using lycopene containing 97.8% *Z*-isomers, uniformly sized particles of an average size of 75 nm were obtained (Figure 3). The reason why nanoparticles were successfully formed from *Z*-isomers is due to the low crystallinity compared with the all-*E*-isomer. In addition, little has been reported on carotenoid micronization using RESS precipitation: the substance, which must be reduced in size, is dissolved in pure SC-CO_2_ and then the solution is suddenly depressurized through a nozzle and expands inside a chamber under lower pressure. This would be because carotenoids have extremely low solubility in pure SC-CO_2_. However, as *Z*-isomers of carotenoids have relatively high solubility in SC-CO_2_ [18,19,21,84], the *Z*-isomers would successfully form nano-sized particles by RESS precipitation, representing a micronization method without the use of organic solvents.

### 5.3. Improvement of Carotenoid Emulsification

**Figure 4:**
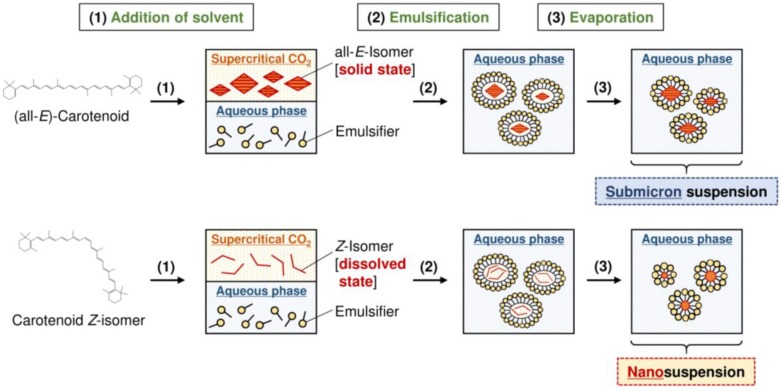
Schematic chart showing preparation of β-carotene suspensions by emulsification–evaporation technique with SC-CO_2_, using (all-*E*)-β-carotene and β-carotene *Z*-isomers as the raw materials [20].

In recent years, as carotenoids are safe natural pigments that have health enhancing effects, their demand by the food industry is continuously increasing [126,127]. However, the low water solubility of carotenoids has made their use problematic for food formulations, limiting the favorable effects of carotenoids. Furthermore, the low water solubility of carotenoids reduces their bioavailability [128,129]. Therefore, improved dispersibility in water by emulsification is very important for the food industry and acts to increase their bioavailability. It is preferred that the suspended preparation contains nano-sized particles for higher dispersibility and bioavailability [111,130]. To obtain nanosuspensions of carotenoids, the following emulsification–evaporation technique is frequently used [131,132,133]: (1) Dissolution of carotenoids in an organic phase; (2) Distribution processing of the solution with water containing an emulsifier; (3) Solvent evaporation under reduced pressure. In this technique, it is important to select an appropriate distribution processing method, e.g., ultrasound treatment, high-speed homogenization, high-pressure homogenization, and microfluidizer treatment [131,132,133,134,135,136]. In addition, the selection of a solvent that can dissolve the target carotenoid is also a very important factor to efficiently produce carotenoid emulsions. However, since the degree of carotenoid solubility in safe and sustainable solvents, such as ethanol and supercritical SC-CO_2_, is very low [14,18,19,21,84], toxic solvents are used in many cases. To improve the emulsification efficiency of carotenoids using the sustainable solvent SC-CO_2_, Ono et al. (2018) [20] focused on increased carotenoid solubility in solvents by *Z*-isomerization. Namely, they investigated the impact of *Z*-isomer content on the production of β-carotene suspensions by the emulsification–evaporation technique. As the organic phase, they used SC-CO_2_ (Figure 4). When β-carotene rich in *Z*-isomers (79.1% of total β-carotene) was used as the raw material, the encapsulated β-carotene content was notably increased compared with the all-*E*-isomer. For example, the encapsulated β-carotene content was 21.2 times higher after emulsification treatment by ultrasound at 45 kHz for 60 min. In addition, when (all-*E*)-β-carotene was used as the raw material, the mean particle size of the obtained suspension was approximately 700 nm, whereas that of β-carotene rich in *Z*-isomers was approximately 100 nm. Thus, *Z*-isomerization treatment before distributed processing is effective for the preparation of carotenoid suspensions by the emulsification–evaporation technique. However, the storage stability of a *Z*-isomer-rich β-carotene suspension was lower than that of all-*E*-isomer-rich one, possibly due to increases in the contact area with oxygen as the particle size decreased [20]. For practical application of this suspension preparation technique, establishment of a method to increase the storage stability of carotenoid *Z*-isomers is essential.

## 6. Conclusions and Future Perspectives

**Figure 5:**
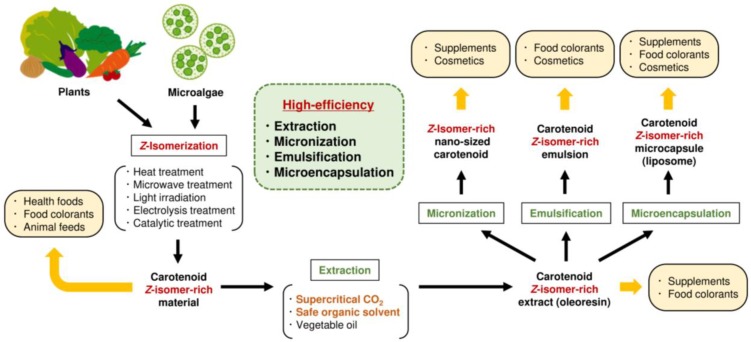
Increased efficiency of carotenoids processing by *Z*-isomerization and applications of *Z*-isomer-rich carotenoids materials.

This review summarizes alterations in the physicochemical properties (color value, solubility, crystallinity, melting point, and stability) of carotenoids by *Z*-isomerization and their application for carotenoid processing (extraction, micronization, and emulsification), specifically using a green and sustainable solvent—SC-CO_2_—and presents typical *Z*-isomerization methods and the effect of *Z*-isomerization on the bioavailability and functionality of carotenoids. As the method for *Z*-isomerization of carotenoids, heat treatment, microwave treatment, light irradiation, electrolysis treatment, and catalytic treatment have been well reported. Since these *Z*-isomerization methods have several advantages and disadvantages, it is necessary to select the appropriate *Z*-isomerization method according to the circumstances. Ample studies have demonstrated that *Z*-isomerization of carotenoid affected the bioavailability, antioxidant capacity, and functionalities such as anticancer activity and antiinflammatory activity and often offered positive impacts on human. The Z-isomerization also induces changes in the physicochemical properties of carotenoids, such as solubility and crystallinity. Namely, the solubility in organic solvents, SC-CO_2_, and oils dramatically is enhanced and crystallinity is reduced by *Z*-isomerization. Since the (all-*E*)-carotenoid, which is a predominant isomer in plants and synthetic ingredients, has very low solubility in SC-CO_2_, its industrial use in carotenoid processing faces a very high hurdle. However, it is highly expected that this impediment could be improved by utilizing the alterations in physicochemical properties of carotenoids by *Z*-isomerization. Carotenoid processing utilizing *Z*-isomerization and the expected application of *Z*-isomer-rich carotenoid materials are summarized in Figure 5. Plants and microalgae rich in carotenoid *Z*-isomers would be applicable as raw materials for the efficient extraction of carotenoids using solvents such as SC-CO_2_, for use in health foods, food colorants, and animal feed. The obtained extract rich in carotenoid *Z*-isomers is expected to be applied to the production of supplements and food colorants with high carotenoid bioavailability and functionality. When safe and sustainable extraction solvents, such as ethanol and supercritical CO_2_, are employed, the value of the extract is anticipated to increase. Furthermore, utilization of carotenoid *Z*-isomer-rich extracts as the raw material is expected to increase the production and quality of nano-sized carotenoids and carotenoid emulsions. The obtained nano-sized carotenoids and carotenoid emulsions rich in *Z*-isomers are expected to be utilized as supplements, food colorants, and cosmetics. In addition, alterations in the physicochemical properties of carotenoids by *Z*-isomerization may be beneficial for the production of microcapsules prepared using carotenoid-containing liposomes. The studies on increasing efficiency of carotenoid processing by *Z*-isomerization pretreatment has just started in recent years. Thus, there is still considerable room for the development of this research field. Fundamental study of this technology will be actively conducted in the future and practical applications are expected.

## Figures and Tables

**Table 1:** Summary of representative methods for *Z*-isomerization of carotenoids and their advantages and disadvantages.

Method	Reported Carotenoid	Advantage	Disadvantage	Reference
Heat treatment	Lycopene, β-carotene, astaxanthin, lutein, etc.	• Simple and conventional method • Requires minimal amount of additive	• Can cause thermal degradation	[12,21,26,27,28]
Microwave treatment	Lycopene, β-carotene, astaxanthin, lutein, etc.	• Simple and fast (few minutes) method • Requires minimal amount of additive	• Can cause thermal degradation • Difficult to control final product quality • High cost of instrumentation	[29,30,31,32]
Light irradiation	Lycopene, β-carotene, lutein, etc.	• Rapid method • Non-thermal process • Low energy usage	• Can cause light degradation • Need to add photosensitizers • Need to remove photosensitizers if toxic ones used • High cost of some photosensitizers	[26,33,34,35,36]
Electrolysis treatment	β-Carotene, 8′-apo-β-caroten-8′-al, canthaxanthin	• Simple and highly efficient method • Non-thermal process • Can prevent degradation during processing	• High cost of instrumentation • Need to remove electrolyte substances if toxic ones used	[37,38]
Catalytic treatment	Lycopene, β-carotene, astaxanthin, zeaxanthin, etc.	• Simple and highly efficient method • Can prevent degradation during the processing • Low energy usage	• Need to remove catalysts if toxic ones used • Can promote degradation in some catalysts • High cost of some catalysts	[29,39,40,41,42,43,44,45,46]

**Table 2:** Comparison of the bioavailability and functionality of all-*E*- and *Z*-isomers of carotenoids.

Carotenoid	Bioavailability	Antioxidant Capacity	Other Functionality
Lycopene	• *E* ^a^ < *Z* ^b^ (Oral study in humans) [22,50] • *E* < *Z* (Oral study in ferrets) [51] • *E* < *Z* (Caco-2 cell model) [52] • *E* < *Z* (Diffusion model) [53]	• *E* ≤ *Z* (TEAC assay) [24,64] • *E* < *Z* (LPSC assay) [24] • *E* ≤ *Z* (MbFe^III^-LP assay) [24] • *E* ≈ *Z* (FRAP assay) [24]	Antiobesity activity: • *E* ≈ 5*Z* (Adipocyte model) [80]
α-Carotene	–	• 13′*Z* > *E* ≈ 9′*Z* > 9*Z* ≈ 13*Z* (TEAC assay) [64]	–
β-Carotene	• *E* > 9*Z* (Oral study in humans) [54,55,56,57,58] • *E* > *Z* (Oral study in ferrets) [59] • *E* > *Z* (Oral study in gerbils) [60] • *E* > *Z* (Caco-2 cell model) [61] • *E* < *Z* (Digestion model) [81]	• *E* < *Z* (Oral study in rats) [65] • *E* ≈ *Z* (TEAC assay) [64] • *E* ≈ 9*Z* ≈ 13*Z* > 15*Z* (TEAC assay) [66] • *E* ≈ *Z* (FRAP assay) [66] • *E* ≈ 9*Z* ≈ 13*Z* > 15*Z* (CL assay) [66]	Antiatherogenesis activity: • *E* < 9*Z* (Oral study in mice) [71] Antiatherosclerosis activity: • *E* < 9*Z* (Oral study in mice) [72,73]
Astaxanthin	• *E* ≤ *Z* (Oral study in humans) [23] • *E* < *Z* (Caco-2 cell model) [25] • *E* < *Z* (Digestion model) [25]	• *E* < *Z* (DPPH assay) [46,67] • *E* < *Z* (ORAC assay) [46] • *E* < *Z* (PLC assay) [46] • *E* < *Z* (Enzyme activity assay) [25] • *E* < *Z* (Lipid- peroxidation assay) [67]	Antiinflammatory activity: • *E* < *Z* (Caco-2 cell model) [76] Antiaging activity: • 9*Z* > *E* > 13*Z* (*Caenorhabditis elegans* model) [79]
Canthaxanthin	–	• *E* < 9*Z* (DPPH assay) [68] • *E* < 9*Z* (Fluorescence assay) [68]	Proapoptotic activity: • *E* < 9*Z* (THP-1 macrophage model) [74]
Fucoxanthin	–	• *E* < *Z* (DPPH assay) [69] • 13*Z* and 13′*Z* > *E* > 9′*Z* (DPPH assay) [70] • 13*Z* and 13′*Z* > *E* > 9′*Z* (Superoxide-detection assay) [70] • 9′*Z* > *E* > 13*Z* and 13′*Z* (ABTS assay) [70] • 9′*Z* > *E* > 13*Z* and 13′*Z* (Hydroxyl radical-scavenging assay) [70]	Anticancer activity: • *E* < *Z* (Caco-2 cell model) [75] • *E* < *Z* (HL-60 cell model) [75]
Lutein	• *E* > *Z* (Caco-2 cell model) [62] • *E* < *Z* (Digestion model) [62]	• *E* < *Z* (FRAP assay) [62] • 13′*Z* > *E* ≈ 9*Z* (DPPH assay) [62] • 13′*Z* > *E* ≈ 9*Z* (ORAC assay) [62] • *E* ≈ *Z* (CAA assay) [62]	–
Zeaxanthin	–	• *E* ≈ 13*Z* > 9*Z* (TEAC assay) [64]	–

^a^ all-*E*-isomer of carotenoid. ^b^ *Z*-isomer of carotenoid.

**Table 3:** Differences in physicochemical properties between (all-*E*)-carotenoids and *Z*-isomers.

Color Value	Solubility	Crystallinity	Melting Point	Stability
*E*^a^ > *Z* ^b^	*E* < *Z*	*E* > *Z*	*E* > *Z*	*E* > *Z*

^a^ all-*E*-isomer of carotenoid. ^b^ *Z*-isomer of carotenoid.
